# Spatial analysis of human *Coxiella burnetii* infection and populations of goat and cattle in Korea, 2015-2024

**DOI:** 10.4178/epih.e2025068

**Published:** 2025-12-09

**Authors:** Seung-Bum Kang, Dae Sung Yoo

**Affiliations:** Department of Veterinary Epidemiology, College of Veterinary Medicine, Chonnam National University, Gwangju, Korea

**Keywords:** *Coxiella burnetii*, Goats, Spatial analysis, Q fever, Zoonoses

## Abstract

**OBJECTIVES:**

Q fever is a bacterial zoonosis that occurs worldwide. Although several studies have reported associations between goat populations and human Q fever outbreaks in Korea, spatial correlation analyses remain limited. Therefore, this study examined the geographic correlation between human Q fever outbreaks and the distributions of goats and cattle in Korea.

**METHODS:**

This study covered a 10-year period (2015-2024), using each of the 250 districts in Korea as the unit of analysis. Data were divided into 2 time periods: 2015-2019 and 2020-2024. Hotspots for the standardized incidence ratio (SIR) were identified using Getis-Ord Gi*. Spatial correlations between SIR and goat and cattle populations were evaluated using a multivariable spatial error model, and the associations between hotspot variables and livestock abundance were assessed using a multivariable Leroux conditional autoregressive model.

**RESULTS:**

SIRs for human Q fever showed significant positive spatial associations with goat populations in 2016 (coefficient=46.52, p<0.01) and 2021 (coefficient=70.97, p<0.01). The associations between goat populations (2016 and 2021) and hotspot classifications were consistent across both periods, with the odds ratio increasing from 1.87 (95% credible interval [CrI], 1.23 to 2.85) in 2015-2019 to 2.33 (95% CrI, 1.55 to 3.64) in 2020-2024. No significant associations were observed between human Q fever and cattle populations.

**CONCLUSIONS:**

Goat populations are becoming more strongly spatially correlated with human Q fever incidence. These findings underscore the need for enhanced preventive management of goat farms to mitigate future outbreaks.

## GRAPHICAL ABSTRACT

[Fig f4-epih-47-e2025068]

## Key Message

This is the first study to spatially study the association of goats and cattle with human outbreak of Q Fever in Korea. The spatial correlation between SIRs and hotspot classification of human Q fever, and goat population, was found to be statistically significant during the period 2015 to 2024. However, the cattle population did not show a significant correlation. The implication is that health authorities need to educate and manage goat farmers to manage human outbreaks of Q fever.

## INTRODUCTION

Extensive epidemiological surveys have confirmed the near-global presence of Q fever, with the notable exceptions of Antarctica and New Zealand [1,2]. *Coxiella burnetii*, an obligate intracellular bacterium, is the causative agent of the zoonotic disease Q fever. *C. burnetii* is especially noteworthy for its ability to form spore-like structures that promote prolonged environmental survival [3,4]. Cattle, sheep, and goats constitute the primary animal reservoirs responsible for transmitting Q fever to humans [5]. Livestock may develop reproductive disorders and can shed *C. burnetii* through birth fluids, feces, and milk, especially during parturition [6].

Q fever is primarily transmitted to humans through inhalation of contaminated aerosols, including dried fecal material, or through direct contact with secretions from infected animals. Less commonly, transmission occurs via ingestion of contaminated milk, person-to-person contact, or sexual contact [7,8]. Wind plays a particularly important role in airborne spread, with rural areas experiencing peak *C. burnetii* exposure risk within 5 kilometers of potential sources [9,10]. Although *C. burnetii* has been detected in approximately 140 tick species, tick-borne transmission to humans appears to be rare [11].

The principal clinical manifestations of acute Q fever are pneumonia, hepatitis, and fever, which may occur independently. Additional complications, such as meningitis, pericarditis, and myocarditis, develop in approximately 1% of acute cases [12]. Endocarditis is the most common presentation of chronic Q fever, especially among individuals with underlying valvular disease [8,13].

In Korea, *C. burnetii* has been detected in a wide range of domestic and wild animals, including cattle, goats, pigs, horses, dogs, cats, wild boars, Korean water deer, wild rodents, and ticks [14-22]. Performing carcass evisceration in slaughterhouses and conducting pre-slaughter inspections of cattle, goats, or sheep in veterinary laboratories have been identified as occupational risk factors for *C. burnetii* infection [23,24]. From 2012 to 2021, a statistically significant nationwide correlation was reported between the number of goats and the incidence of human *C. burnetii* infection [25]. Although previous research has demonstrated national-level associations between goat populations and human Q fever cases, spatial analyses that account for geographic variability in livestock distribution remain limited. Therefore, this study aimed to investigate the spatial correlations between human Q fever incidence and the distributions of goats and cattle across districts in Korea.

## MATERIALS AND METHODS

### Study structure

This geographic investigation encompassed the entire Korea. During the study period, the number of administrative cities and districts (*si, gun, gu* [city, county, district]) varied between 252 and 250. Information on Q fever incidence and population characteristics was collected at the administrative district level from January 2015 to December 2024. Because only 250 spatial units existed between 2016 and 2022, all data were converted to align with the 250-unit administrative structure established in 2022.

### Data source

Incidence data for human Q fever were obtained from the Infectious Disease Statistics System of the Korea Disease Control and Prevention Agency [26]. Five cases imported from foreign countries were excluded from the total of 813 occurrences (2015-2024). Human population data were derived from the Population Census of the Korea. To calculate the standardized incidence ratio (SIR) for human Q fever, population counts for 2017 and 2022 were used as denominators [27].

In Korea, no formal animal surveillance program specifically targets Q fever. Non-public data on goat and cattle populations were provided by the Livestock Health Control Association. Goat population data from 2016 and 2021 and cattle population data from 2016 and 2022 were used in the analysis. Due to data constraints, the time periods were not fully aligned. The shapefile for the 2022 administrative map of Korea was obtained from the Statistical Geographic Information Service of Statistics Korea [28].

### Case definition

In Korea, reports of human Q fever are based solely on passive surveillance. Individuals presenting with confirmed pathogen infection or relevant clinical symptoms and epidemiological features were classified as suspected cases and required mandatory reporting. Among these individuals, positive test results from blood culture, indirect immunofluorescence assay, or polymerase chain reaction for gene detection were considered diagnostic [29]. Laboratory confirmation was conducted at the Korea Disease Control and Prevention Agency.

### Statistical analysis

Since 2020, the incidence of respiratory diseases in humans and bovine tuberculosis and brucellosis in animals has shifted following the implementation of non-pharmaceutical interventions during the coronavirus disease 2019 (COVID-19) pandemic [30]. Accordingly, this study analyzed data from 2015 to 2024 by dividing the period into two 5-year intervals (2015-2019 and 2020-2024) to account for unverified confounding factors.

To minimize the influence of differences in regional population distribution on disease risk assessment, district-level SIRs for the first 5-year period (2015-2019) and the second (2020-2024) were used as dependent variables in the spatial regressions. SIRs were calculated for each of the 250 administrative districts. District-level SIRs were computed by dividing the observed number of human Q fever cases by the expected number and multiplying the result by 100 (observed cases/expected cases×100). Expected cases were estimated by multiplying the national incidence rate for the same period (total number of cases in Korea/national population) by the population of each district (expected cases=district population×national incidence rate). This approach follows the principles of indirect standardization, using the national-level risk as the reference. All shapefiles and raster maps used in these procedures were created and processed using QGIS 3.34.15 (https://qgis.org/downloads-list/).

Neighbor relationships were defined using queen contiguity [31]. A Getis-Ord Gi* statistic was applied to identify spatial clusters of districts with high or low SIRs for each period (2015-2019 and 2020-2024) [32,33]. Hotspots were coded as 1, and all other districts were coded as 0. These binary hotspot variables for both periods were then used as dependent variables in the spatial regression analysis.

Spatial autocorrelation refers to the phenomenon in which the values of a variable at one location are systematically related to values of the same variable in neighboring locations, thereby violating the assumption of independence. This spatial dependence weakens the applicability of standard regression models, which assume uncorrelated observations across space.

In this study, global Moran’s I was applied to assess spatial autocorrelation for the district-level SIRs, hotspot classifications, and the residuals of the spatial regression models [34,35]. The interpretation of Moran’s I is straightforward. Values greater than zero indicate positive spatial autocorrelation, meaning that similar values cluster among neighboring units. Values less than zero indicate negative spatial autocorrelation, characterized by dissimilar neighboring values that often appear in an alternating or checkerboard pattern. Values near zero suggest no spatial dependence and reflect spatial randomness in the variable’s distribution. Additional formulas and applications are provided in Supplementary Material 1.

Correcting for spatial autocorrelation is achieved through the use of a spatial error model (SEM). The SEM incorporates a function of the unexplained error term and the errors of neighboring units. A multivariable SEM was constructed to examine the relationships between human Q fever occurrences and goat and cattle populations. The district-level SIRs for 2015-2019 and 2020-2024 served as the response variables. Goat populations in 2016 and 2021 and cattle populations in 2016 and 2022 were used as explanatory variables for each corresponding period. Maximum likelihood estimation was employed for the SEM.

The SEM is typically presented as 2 equations [36]:


y=α+Xβ+ε, ε=λWε+μ

In this model, α denotes the intercept, λ represents the spatial autoregressive parameter, and W is an n×n spatial weights matrix. The vectors y and ε are n×1 and represent, respectively, observations of the dependent variable and latent error terms. X is a matrix of explanatory variables. The error vector μ follows a normal distribution, μ~N(0, σ²I).

A multivariable logistic regression model incorporating spatial random effects via a Leroux conditional autoregressive (CAR) prior was used to analyze the correlations between hotspots and the populations of goats and cattle. The hotspots of the human Q fever SIRs for 2015-2019 and 2020-2024 were treated as binary response variables. Goat populations from 2016 and 2021 and cattle populations from 2016 and 2022 were standardized and used as explanatory variables for each period. The model specifies a binomial likelihood with a logistic link function, expressed as follows [37]:


$$\ln\left(\frac{P\left(Z=1|X_{1},\ldots ,X_{k}\right)}{1-P\left(Z=1|X_{1},\ldots ,X_{k}\right)}\right)=\beta_{0}+\overset{p}{\underset{k=1}{\sum }}\beta_{k}X_{k}+u_{i}$$

In this equation, Z denotes the binary outcome, X=(X₁, …, X_p_) represents the explanatory variables, βₖ are their corresponding coefficients, and uᵢ is the spatially structured random effect for areal unit *i*. The spatial random effects uᵢ are modeled using the Leroux CAR prior. This specification includes a single spatial random effect, where the degree of spatial autocorrelation among neighboring areal units is governed by a constant parameter ρ, resulting in the following formulation [38]:


$$\left(u_{i}|u_{j},i\neq j,\tau_{u}^{2}\right)∼N\left(\frac{\rho \sum_{j}u_{j}\omega_{ij}}{\rho \sum_{j}\omega_{ij}+1-\rho },\frac{\tau_{u}^{2}}{\rho \sum_{j}\omega_{ij}+1-\rho }\right)$$

Here, ωᵢⱼ indicates whether regions *i* and *j* are adjacent, and $\tau_{u}^{2}$, the marginal variance of the spatial effect, is given an inverse-gamma prior $\tau_{u}^{2}$~IG(1, 0.01). Spatial dependence is controlled by ρ {0, 1}, which is assigned a uniform(0, 1) prior. When ρ=1, the prior simplifies to the intrinsic CAR model, representing strong spatial dependence, whereas when ρ=0, the model reflects spatially independent random effects.

Model estimation was conducted within a Bayesian framework, using 3,000,000 samples generated from 4 independent Markov chain Monte Carlo (MCMC) chains. A burn-in of 300,000 iterations and a thinning interval of 300 were applied. The 95% credible intervals (CrI) were calculated using the 2.5th percentile and 97.5th percentile of the posterior distribution based on the retained samples. Spatial analyses were performed using the CARBayes package [38].

Data management and statistical analyses were conducted using GeoDa 1.22 and R version 4.4.3 (R Foundation for Statistical Computing, Vienna, Austria).

### Ethics statement

Due to the lack of personally identifiable health information in our data, ethical approval was not required.

## RESULTS

Excluding 5 cases that were not of domestic origin, a total of 808 human Q fever cases were reported in Korea between January 2015 and December 2024. The incidence rates of human Q fever from 2015 to 2019 and from 2020 to 2024 were 0.20 cases per 100,000 person-years and 0.10 cases per 100,000 person-years, respectively. In 2016, the district-level populations of goats and cattle were 0 to 13,761 heads and 0 to 116,625 heads, respectively. In 2021, the district-level goat populations were 0 to 11,925 heads, and in 2022, the district-level cattle populations were 0 to 104,624 heads (Table 1).

**Figure 1.**
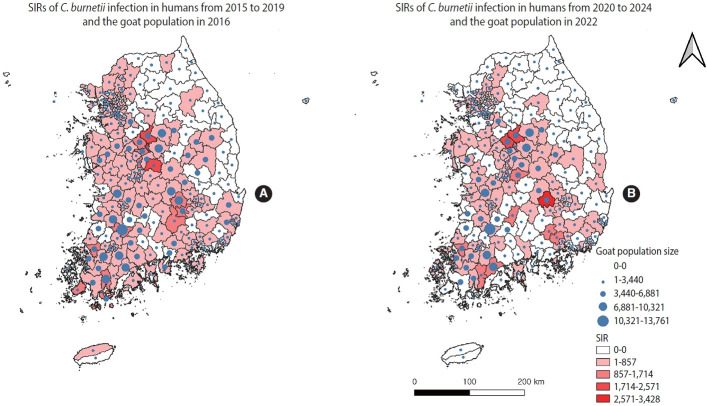
Geographical distribution of area-specific standardized incidence ratio (SIR) of *Coxiella burnetii* infection in humans and distribution of the goat population in Korea. (A) SIRs of *C. burnetii* infection in humans from 2015 to 2019 and the goat population in 2016. (B) SIRs of *C. burnetii* infection in humans from 2020 to 2024 and the goat population in 2022. Goat population size is shown as a function of the centroid size of each spatial unit, and SIR values are represented by the color of each spatial unit.

**Figure 2.**
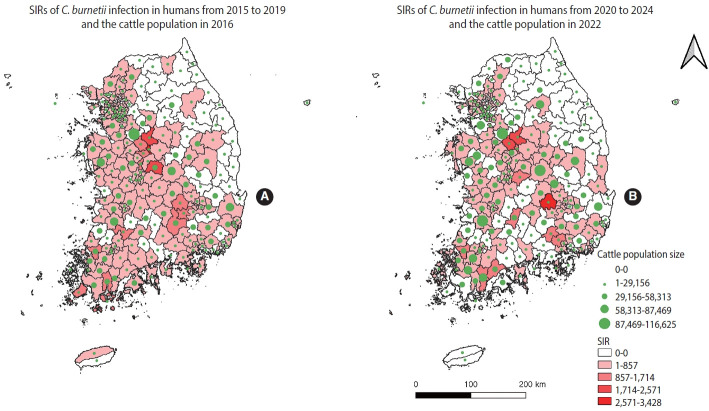
Geographical distribution of area-specific standardized incidence ratio (SIR) of *Coxiella burnetii* infection in humans and distribution of the cattle population in Korea. (A) SIRs of *C. burnetii* infection in humans from 2015 to 2019 and the cattle population in 2016. (B) SIRs of *C. burnetii* infection in humans from 2020 to 2024 and the cattle population in 2022. Cattle population size is shown as a function of the centroid size of each spatial unit, and SIR values are represented by the color of each spatial unit.

The SIRs of human Q fever, along with goat and cattle population counts during the study period, are visualized in Figures 1 and 2, respectively.

### Spatial analyses

Table 2 presents the spatial regression results for district-level SIRs of human Q fever and the distributions of goat and cattle populations in Korea for 2015-2019 and 2020-2024. During the 2015-2019 period, district-level SIRs were positively correlated with goat population distribution in 2016 (coefficient=46.52, p<0.01). However, the spatial correlation with cattle population distribution in 2016 was not statistically significant (p=0.35). A stronger positive spatial correlation was observed between the 2020-2024 SIRs and the 2021 goat population distribution (coefficient=70.97, p<0.01), indicating an increase in the strength of association across periods. In contrast, the spatial correlation with cattle population distribution in 2022 remained statistically insignificant (p=0.9). The Akaike information criterion values for the 2 study periods were 3,605.5 and 3,596.1, providing measures of relative model quality.

**Figure 3.**
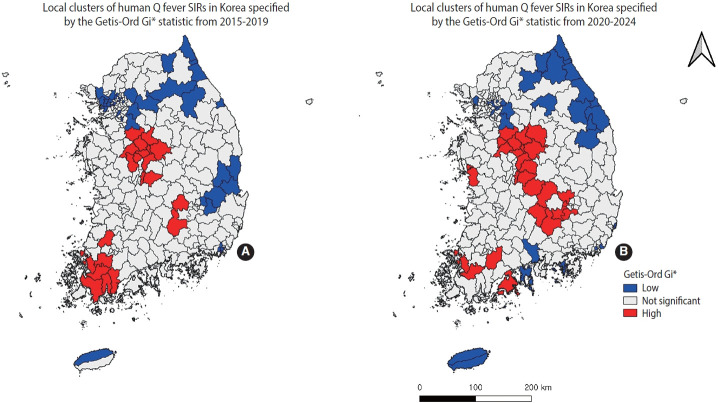
Cluster maps of standardized incidence ratios (SIRs) for human Q fever in Korea, 2015-2024 (p<0.05). (A) Local clusters of human Q fever SIRs in Korea specified by the Getis-Ord Gi* statistic from 2015-2019. (B) Local clusters of human Q fever SIRs in Korea specified by the Getis-Ord Gi* statistic from 2020-2024.

Figure 3A and B illustrate the clustering patterns of positive and negative spatial autocorrelation in human Q fever, as identified by the Getis-Ord Gi* statistic for 2015-2019 and 2020-2024, respectively. Both periods exhibited heterogeneous clusters.

During 2015-2019, 21 regions with high SIRs (hotspots) and 42 regions with low SIRs (cold spots) were identified (p<0.05). The hotspots included Daejeon (1), Sejong (1), Gyeonggi (1), North Chungcheong (8), South Chungcheong (1), South Jeolla (7), North Gyeongsang (1), and South Gyeongsang (1). The cold spots included Seoul (7), Busan (7), Daegu (1), Incheon (5), Gyeonggi (9), Gangwon (7), North Gyeongsang (5), and Jeju (1). Parentheses indicate the number of sub-regional units within each district.

During 2020-2024, 33 hotspots and 25 cold spots were detected (p<0.05). The hotspots included Daejeon (1), Gyeonggi (1), North Chungcheong (10), South Chungcheong (3), South Jeolla (4), South Gyeongsang (2), Daegu (1), and North Gyeongsang (3). Cold spots included Seoul (5), Busan (3), Ulsan (1), Incheon (1), Gyeonggi (5), Gangwon (12), South Jeolla (1), North Gyeongsang (1), South Gyeongsang (2), and Jeju (2). Detailed geographic information for both periods appears in Supplementary Material 2.

Standard MCMC diagnostics (Table 3) indicated good mixing and convergence, with effective sample sizes of 35,000-38,000, acceptance rates of 44-48%, and potential scale reduction factor (PSRF) upper bounds of 1. Table 3 summarizes the results of spatial logistic regression models using the Leroux CAR prior with a logit link function, in which hotspot regions were coded as 1 and all other regions as 0.

Across the 10-year study period, human Q fever hotspots were significantly and positively associated with goat population densities. For 2015-2019, a 1-standard-deviation increase in goat population (2016) corresponded to an 87% increase in the odds of classification as a hotspot (odds ratio [OR], 1.87), controlling for other variables. For 2020-2024, a 1-standard-deviation increase in goat population (2021) corresponded to more than a twofold increase in hotspot classification (OR, 2.33). In both periods, the 95% CrI for these ORs were entirely above 1.00, providing strong statistical evidence for positive associations.

A positive spatial association was also observed between human Q fever hotspots and cattle distribution, with ORs of 1.52 (2016) and 1.18 (2022) for the 2 periods. However, the 95% CrI for cattle effects—0.98 to 2.32 (2016) and 0.73 to 1.83 (2022)—were wide and included 1.00, indicating substantial uncertainty and a lack of definitive statistical significance.

The estimated spatial variance τ² remained below 0.01 with narrow CrI, suggesting minimal unexplained local spatial variation. The spatial autocorrelation parameter ρ was consistently around 0.75 across models, indicating moderate to strong spatial dependence in the random effects.

The district-level SIRs of human Q fever exhibited spatial autocorrelation in both study periods (Table 4). For 2015-2019, global Moran’s I was positive with a large Z-value and p<0.01, indicating significant positive global spatial autocorrelation. The same pattern was observed for 2020-2024, with Moran’s I remaining positive and statistically significant (p<0.01).

Following SEM-based spatial regression for both periods, Moran’s I values computed from the residuals were near zero, with low Z-values and non-significant p-values, suggesting negligible residual spatial structure (Table 4). These findings indicate that the SEM effectively captured the spatial correlation present in the SIR data.

For the hotspot variable, significant positive Moran’s I values with large Z-statistics were identified for both periods (p<0.01), demonstrating strong spatial autocorrelation. After applying the Leroux CAR model, the residual Moran’s I remained positive and statistically significant in both periods (p<0.01), though with reduced magnitudes and Z-values compared to the pre-model statistics. This pattern indicates that while the Leroux CAR model weakened the spatial dependence, it did not fully account for the underlying spatial structure of the hotspot variable.

## DISCUSSION

This study identified statistically significant and positive associations between goat population and human Q fever—measured by both SIRs and hotspot classifications—during the periods 2015-2019 and 2020-2024, whereas no significant associations were observed with cattle population. At the district level, human Q fever SIRs showed a significant positive spatial association with goat population in 2016 (coefficient=46.52, p<0.01) and 2021 (coefficient=70.97, p<0.01), with the latter period (2020-2024) demonstrating a stronger association. In contrast, no significant spatial correlations were identified with cattle populations in 2016 (p=0.35) or 2022 (p=0.90). This spatial–statistical pattern aligns with Korean evidence documenting that improper disposal of postpartum animal tissues at a goat farm caused widespread contamination, serving as a principal source of human infection [39].

A comparison of the 2 periods revealed a marked increase in the strength of associations between goat population (2016 and 2021) and hotspot classification, with the OR rising from 1.87 (95% CrI, 1.23 to 2.85) in 2015-2019 to 2.33 (95% CrI, 1.55 to 3.64) in 2020-2024. By contrast, the 95% CrIs for the association between human Q fever hotspots and cattle distribution—0.98 to 2.32 (2016) and 0.73 to 1.83 (2022)—included 1.00 in both periods, indicating considerable uncertainty. From 2015 to 2023, the number of goats per farm generally increased, followed by a moderating trend in 2023 [40]. This pattern may have influenced Q fever transmission within farms and among nearby residents, potentially contributing to the stronger associations observed in the later period. The explanatory power of the Leroux CAR model is limited: although it attenuated spatial autocorrelation, it did not fully remove residual dependence. A key constraint relates to residence-based geocoding of national Q fever notifications, which can misclassify livestock-related exposures when infections occur in occupational or non-residential settings. This residence–exposure mismatch weakens fixed effects, shifts unexplained variation into the spatial random component, and leaves persistent residual spatial structure.

The association between human Q fever and goat population may reflect elevated *C. burnetii* infection risk among individuals living near goat farms, a risk driven by continuous environmental contamination during abortion events and the prolonged persistence of pathogens around farms. Previous research supports this interpretation: individuals residing within 2 km of large affected dairy goat farms (i.e., >400 animals) have significantly higher Q fever risk than those living more than 5 km away [41]. Experimental studies have shown that Q fever–induced abortions in pregnant goats can occur at rates up to 90% [42]. After abortion, bacterial shedding has been detected in vaginal secretions for up to 14 days and in milk for up to 52 days [42]. Moreover, parturition in infected goat herds represents a major route for environmental contamination, with the bacterium capable of surviving in the farm environment for at least 1 year [43].

These findings are consistent with previous international research documenting positive associations between goat populations and human Q fever incidence. Elevated human seroprevalence has been reported in areas with high goat seropositivity and documented goat abortions, as demonstrated in studies from Cyprus, Pakistan, and the Netherlands [44]. Similarly, fluctuations in human Q fever incidence in Taiwan between 2004 and 2012 were more strongly correlated with goat population dynamics than with cattle [45].

One limitation of the present study is the absence of livestock surveillance data for Q fever, which prevents direct comparisons between human and animal outbreak patterns. Addressing this gap will require implementation of integrated livestock surveillance that is spatially and temporally linked to human case notifications.

As kidding in Korean native black goats is concentrated in cooler seasons (fall and spring), the finding of substantial heritability in reproductive timing is biologically relevant [46]. During these periods, environmental loading and aerosolization of birthing products may increase, as may animal handling and transport, thereby creating more opportunities for human exposure. Additionally, considering seasonal meteorological factors—such as wind speed, temperature, and precipitation—would help clarify conditions that facilitate airborne transmission around goat farms in Korea [39].

In conclusion, this study confirms that the goat population is a key risk factor for human Q fever and highlights the need for targeted public health interventions. Policy decisions should incorporate the risks associated with goat farming, and practical measures—such as improved farm biosecurity, enhanced hygiene, and strengthened public education—are essential, particularly for high-risk communities. From an academic standpoint, the findings underscore the importance of advanced spatial modeling and a One Health perspective.

## Figures and Tables

**Figure f4-epih-47-e2025068:**
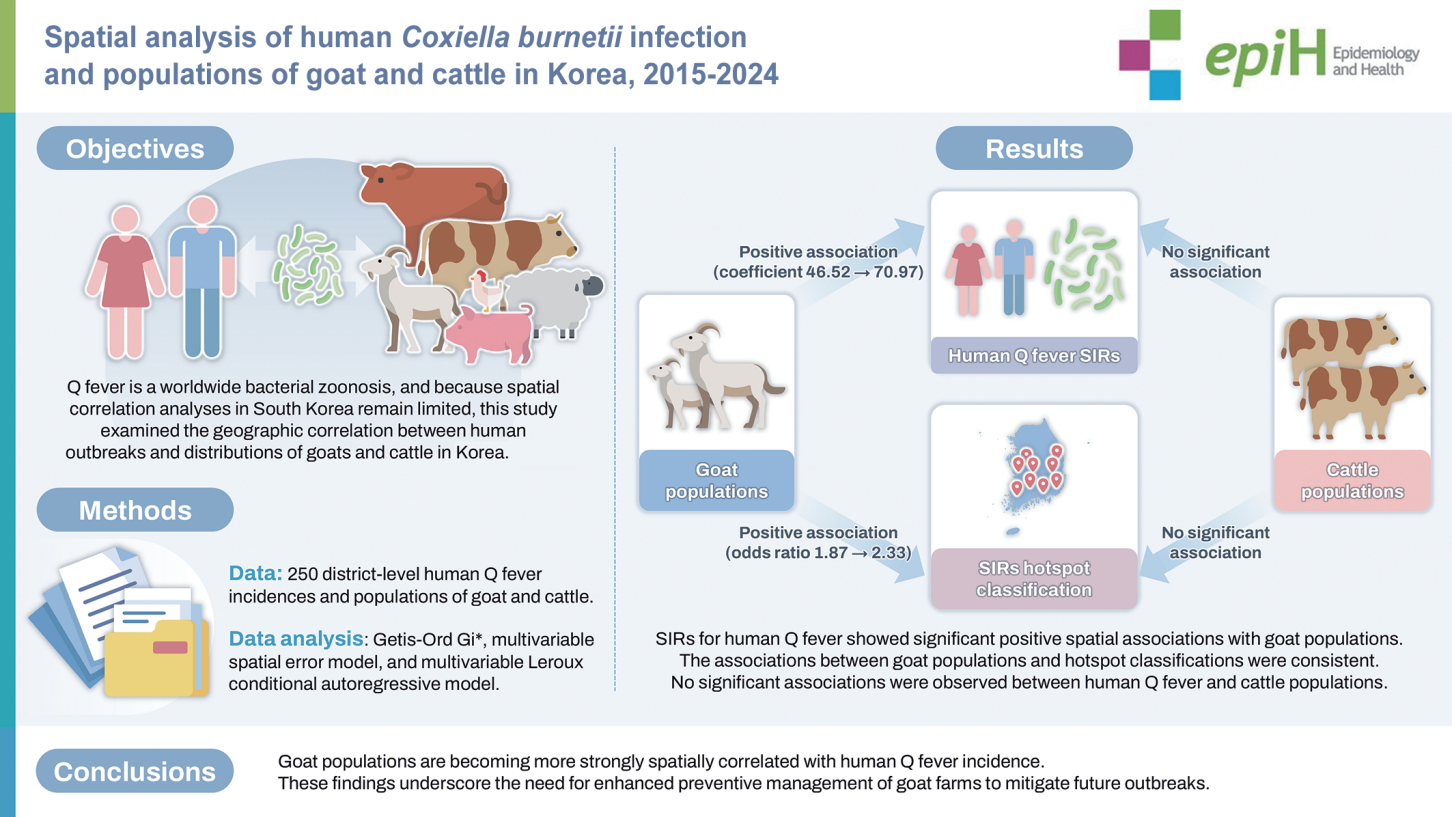


**Table 1.** Descriptive statistics of human and livestock populations and SIRs of Coxiella burnetii (Q fever) cases in humans, across 250 administrative districts in Korea

Statistics	Human population		SIRs		Goat population		Cattle population	
	2017	2022	2015-2019	2020-2024	2016	2021	2016	2022
Scale	1 Person	1 Person	-	-	1,000 Heads	1,000 Heads	1,000 Heads	1,000 Heads
Mean	205,690	206,769	187	181	1.79	1.46	13.79	16.26
Median	172,683	176,321	53.4	38.9	0.90	0.67	6.37	7.82
SD	163,037	168,815	374	358	2.33	1.97	17.93	20.92
Min	8,702	8,288	0	0	0	0	0	0
Max	845,514	931,472	3,428	2,712	13.76	11.92	116.62	104.62

SIR, standardized incidence ratios; SD, standard deviation; Min, minimum; Max, maximum.

**Table 2.** Geographic association of area-specific SIRs^1^ of *Coxiella burnetii* infection in humans and distribution of goat and cattle populations in Korea (2015-2019 and 2020-2024, estimated from multivariable analysis by spatial error model)

Periods	Independent variable^2^	Coefficient	SE	p-value	AIC
2015-2019	Goat population (2016)	46.52	11.80	<0.01	3,605.5
	Cattle population (2016)	-1.36	1.46	0.35	
2020-2024	Goat population (2021)	70.97	13.42	<0.01	3,596.1
	Cattle population (2022)	0.16	1.26	0.90	

SIR, standardized incidence ratio; SE, standard error; AIC, Akaike information criterion.

1 The area-specific SIR: the observed count of *C. burnetti* cases is divided by the expected count in specific areas and then multiplied by 100.

2 The populations were calculated per 1,000 animals.

**Table 3.** Geographic association of high clusters of area-specific standardized incidence ratio of *Coxiella burnetii* infection in humans by Getis-Ord Gi^1^ and distribution of goat and cattle heads in Korea (2015-2024 and 2020-2024 estimated from multivariable analysis)^2^

Periods	Independent variable	OR (95% CrI)^3^	Mean (95% CrI)
2015-2019	Goat population (2016)^4^	1.87 (1.23, 2.85)	-
	Cattle population (2016)^4^	1.52 (0.98, 2.32)	-
	τ²	-	<0.01 (<0.01, <0.01)
	ρ	-	0.75 (0.51, 0.92)
2020-2024	Goat population (2021)^4^	2.33 (1.55, 3.64)	-
	Cattle population (2022)^4^	1.18 (0.73, 1.83)	-
	τ²	-	<0.01 (<0.01, <0.01)
	ρ	-	0.75 (0.51, 0.92)

OR, odds ratio; CrI, credible interval.

1 The hotspots identified by Getis-Ord Gi* and the other regions are transformed into a binary variable, usually coded as 1 for the hotspots and 0 for the rest of the regions.

2 Model parameters of variables were estimated using the Leroux conditional autoregressive prior model with logit link function with spatial weight matrix where the neighborhood was defined as the area sharing a border; Diagnostics: four chains; effective sample sizes approximately 35,000-38,000 for most parameters; chain acceptance rates 44-48%; potential scale reduction factor (95% upper confidence bound)=1.00 for all parameters in both periods.

3 Obtained by exponentiating the estimated coefficients, i.e., *e*β.

4 Calculated per 1,000 animals and standardized using a Z-score transformation.

**Table 4.** Global Moran’s I of human Q fever with and without spatial regression in 250 districts of Korea, 2015-2019 and 2020-2024^1^

Data types	Periods	Global Moran’s I	SD	p-value
SIR of human Q fever	2015-2019	0.34	8.78	<0.01
	2020-2024	0.26	6.60	<0.01
Hotspot variable by SIR of human Q fever	2015-2019	0.47	11.68	<0.01
	2020-2024	0.38	9.35	<0.01
Residuals from the SEM	2015-2019	0.00	0.01	0.42
	2020-2024	0.00	0.21	0.49
Residuals from the Leroux CAR model	2015-2019	0.37	9.20	<0.01
	2020-2024	0.24	6.08	<0.01

SD, standard deviation; SIR, standardized incidence ratio; SEM, spatial error model; CAR, conditional autoregressive.

1 For all results shown in the table, the expected Moran’s I under spatial randomness was approximately -0.01.

## Supplementary materials

Supplementary Material 1. Methods for Spatial Cluster and Autocorrelation Analysis

Supplementary Material 2. List of regions identified as spatial hot spots and cold spots based on Standardized Incidence Ratios (SIRs), 2015–2019 and 2020–2024.
