# Supplementary material for: Spatial analysis of human Coxiella burnetii infection and populations of goat and cattle in Korea, 2015-2024
Source: Epidemiol Health. 2025 Dec 9;47:e2025068. doi: 10.4178/epih.e2025068 (PMC12884044; doi:10.4178/epih.e2025068)
Supplement: Supplementary Material 1. — Methods for Spatial Cluster and Autocorrelation Analysis [file epih-47-e2025068-Supplementary-1.docx]

Supplementary Material 1. Methods for Spatial Cluster and Autocorrelation Analysis

The spatial weighting matrix employed in this study is of the type known as “Queen Contiguity”. This is a specific form of a binary matrix that utilizes the representation of neighborhood relationships with the digits 1 and 0. In the case of subdistricts that are in close proximity to one another (i.e., contiguous side/angle), the matrix assigns a weight of 1, vice versa.^33^

A Getis-Ord Gi* statistic was employed to investigate the spatial clusters of 250 districts with high or low SIRs in two periods (2015-2019 and 2020-2024)^34, 35^. This metric evaluates the deviation between the observed local sum of Standardized Incidence Ratios (SIRs)—comprising the target district and its geographically adjacent districts—and the expected local sum of SIRs. The Getis-Ord Gi* has the following form: ^35^

$$G_{i}^{*}=\frac{\sum_{j=1}^{n} w_{i,j}x_{j}-\overset{\_}{X}\sum_{j=1}^{n} w_{i,j}}{S\sqrt{\frac{n\sum_{j=1}^{n} w_{i,j}^{2}-{(\sum_{j=1}^{n} w_{i,j})}^{2}}{n-1}}}$$

where x*ⱼ* represents the attribute value for feature *j*, w*ᵢⱼ* indicates the spatial dependence between features i and j, as defined by the spatial weight matrix, and n refers to the total number of features, with:

$$\overset{\_}{X}=\frac{\sum_{j=1}^{n} x_{j}}{n}$$

$$S=\sqrt{\frac{\sum_{j=1}^{n} x_{j}^{2}}{n}-{(\overset{\_}{X})}^{2}}$$

The Gᵢ* statistic is expressed as a z-score. A statistically significant, particularly large, and positive Z-score (p – value < 0.05) indicates a spatially localized cluster of elevated rates (hot spot). Hot spots occur when high-rate districts are clustered together; the observed local SIR sum surpasses the expected sum, and this elevation is beyond what would be predicted by chance. Cold spots, which are indicative of local low-rate clusters, are characterized by statistically significant large negative Z-scores (p – value < 0.05). These are areas where low-rate districts are spatially correlated with neighboring districts with similarly low rates. Both statistically significant hot spots and cold spots were visualized in a 250-district map. Furthermore, the hot spots were defined by the number 1 and the other districts were defined by the number, 0. These two binary variables (2015-2019 and 2020-2024) were used as the dependent variables in the spatial regression analysis.

Global Moran's I was performed for spatial autocorrelation analysis for district-level SIRs of human Q fever for ten years (2015 to 2024). Spatial autocorrelation quantifies the degree to which the observed values of a given variable exhibit non-random spatial structure. Global Moran’s I has the following form:^36^

$$I=\frac{n\sum_{i=1}^{n} \sum_{j=1}^{n} w_{ij}(x_{i}-\overset{\_}{x})(x_{j}-\overset{\_}{x})}{(\sum_{i=1}^{n} \sum_{j=1}^{n} w_{ij})\sum_{i=1}^{n} (x_{i}-\overset{\_}{x})^{2}},i\neq j$$

Where, n is the number of districts, x_i_ is the value at district i, x_j_ is the value at district i's neighbor j, w_ij_ is the spatial weight matrix of districts i and j. The Global Moran’s I statistic takes values within the interval [-1.0, 1.0], with a value of zero reflecting a spatially random distribution of the variable under study, implying the absence of spatial autocorrelation. If SIRs or binary variable of hotspot were positively spatially correlated, the index would be greater than 0, with higher values indicating stronger spatial autocorrelation, and vice versa. Global Moran's I values were calculated for the residuals of all models and compared. This was done to ensure that all spatial dependencies were taken into account.^37^
