# Supplementary material for: Spatial analysis of human Coxiella burnetii infection and populations of goat and cattle in Korea, 2015-2024
Source: Epidemiol Health. 2025 Dec 9;47:e2025068. doi: 10.4178/epih.e2025068 (PMC12884044; doi:10.4178/epih.e2025068)
Supplement: Supplementary Material 2. — List of regions identified as spatial hot spots and cold spots based on Standardized Incidence Ratios (SIRs), 2015–2019 and 2020–2024. [file epih-47-e2025068-Supplementary-2.docx]

Supplementary Material 2. List of regions identified as spatial hot spots and cold spots based on Standardized Incidence Ratios (SIRs), 2015–2019 and 2020–2024.

From 2015 to 2019, 21 regions with high SIRs (hot spots) and 42 regions with low SIRs (cold spots) were detected (*p* < 0.05). The 21 hot-spot regions were: Dong-gu, Daejeon; Sejong-si, Sejong Special Self-Governing City; Anseong-si, Gyeonggi; Sangdang-gu, Cheongju-si, Chungbuk; Heungdeok-gu, Cheongju-si, Chungbuk; Cheongwon-gu, Cheongju-si, Chungbuk; Okcheon-gun, Chungbuk; Jincheon-gun, Chungbuk; Goesan-gun, Chungbuk; Eumseong-gun, Chungbuk; Jeungpyeong-gun, Chungbuk; Dongnam-gu, Cheonan-si, Chungnam; Naju-si, Jeonnam; Jangheung-gun, Jeonnam; Gangjin-gun, Jeonnam; Haenam-gun, Jeonnam; Yeongam-gun, Jeonnam; Muan-gun, Jeonnam; Jangseong-gun, Jeonnam; Seongju-gun, Gyeongbuk; Hapcheon-gun, Gyeongnam

The 42 cold-spot regions were: Jung-gu, Seoul; Gwangjin-gu, Seoul; Seongbuk-gu, Seoul; Eunpyeong-gu, Seoul; Gangseo-gu, Seoul; Guro-gu, Seoul; Yeongdeungpo-gu, Seoul; Jung-gu, Busan; Seo-gu, Busan; Dong-gu, Busan; Busanjin-gu, Busan; Buk-gu, Busan; Yeonje-gu, Busan; Sasang-gu, Busan; Dong-gu, Daegu; Namdong-gu, Incheon; Bupyeong-gu, Incheon; Gyeyang-gu, Incheon; Seo-gu, Incheon; Michuhol-gu, Incheon; Yeongtong-gu, Suwon-si, Gyeonggi; Bucheon-si, Gyeonggi; Deogyang-gu, Goyang-si, Gyeonggi; Namyangju-si, Gyeonggi; Uiwang-si, Gyeonggi; Cheoin-gu, Yongin-si, Gyeonggi; Gimpo-si, Gyeonggi; Gwangju-si, Gyeonggi; Yangpyeong-gun, Gyeonggi; Donghae-si, Gangwon; Sokcho-si, Gangwon; Hongcheon-gun, Gangwon; Pyeongchang-gun, Gangwon; Yanggu-gun, Gangwon; Goseong-gun, Gangwon; Yangyang-gun, Gangwon; Buk-gu, Pohang-si, Gyeongbuk; Yeongcheon-si, Gyeongbuk; Gyeongsan-si, Gyeongbuk; Cheongsong-gun, Gyeongbuk; Yeongdeok-gun, Gyeongbuk; Jeju-si, Jeju

From 2020 to 2024, 33 regions with high SIRs (hot spots) and 25 regions with low SIRs (cold spots) were detected (*p* < 0.05).The 33 hot-spot regions were: Dong-gu, Daejeon; Anseong-si, Gyeonggi; Sangdang-gu, Cheongju-si, Chungbuk; Cheongwon-gu, Cheongju-si, Chungbuk; Okcheon-gun, Chungbuk; Jincheon-gun, Chungbuk; Goesan-gun, Chungbuk; Eumseong-gun, Chungbuk; Jeungpyeong-gun, Chungbuk; Dongnam-gu, Cheonan-si, Chungnam; Yeongam-gun, Jeonnam; Muan-gun, Jeonnam; Hapcheon-gun, Gyeongnam; Dalseong-gun, Daegu; Chungju-si, Chungbuk; Boeun-gun, Chungbuk; Yeongdong-gun, Chungbuk; Seobuk-gu, Cheonan-si, Chungnam; Boryeong-si, Chungnam; Goheung-gun, Jeonnam; Hwasun-gun, Jeonnam; Gimcheon-si, Gyeongbuk; Goryeong-gun, Gyeongbuk; Chilgok-gun, Gyeongbuk; Geochang-gun, Gyeongnam

The 25 cold-spot regions were: Yongsan-gu, Seoul; Seocho-gu, Seoul; Jung-gu, Seoul; Gwangjin-gu, Seoul; Yeongdeungpo-gu, Seoul; Jung-gu, Busan; Busanjin-gu, Busan; Haeundae-gu, Busan; Dong-gu, Ulsan; Seo-gu, Incheon; Sujeong-gu, Seongnam-si, Gyeonggi; Hanam-si, Gyeonggi; Uiwang-si, Gyeonggi; Cheoin-gu, Yongin-si, Gyeonggi; Gwangju-si, Gyeonggi; Donghae-si, Gangwon; Sokcho-si, Gangwon; Yanggu-gun, Gangwon; Goseong-gun, Gangwon; Yangyang-gun, Gangwon; Chuncheon-si, Gangwon; Gangneung-si, Gangwon; Taebaek-si, Gangwon; Samcheok-si, Gangwon; Hoengseong-gun, Gangwon; Jeongseon-gun, Gangwon; Inje-gun, Gangwon; Yeosu-si, Jeonnam; Bonghwa-gun, Gyeongbuk; Tongyeong-si, Gyeongnam; Hadong-gun, Gyeongnam; Jeju-si, Jeju; Seogwipo-si, Jeju
